# Lung development, repair and cancer: A study on the role of *MMP20* gene in adenocarcinoma

**DOI:** 10.1371/journal.pone.0250552

**Published:** 2021-04-29

**Authors:** Pooi Ling Mok, Arun Neela Kumar Anandasayanam, Hernandez Maradiaga Oscar David, Jiabei Tong, Aisha Farhana, Mohammed Safwan Ali Khan, Gothai Sivaprakasam, Avin Ee-Hwan Koh, Badr Alzahrani

**Affiliations:** 1 Department of Clinical Laboratory Sciences, College of Applied Medical Sciences, Jouf University, Sakaka, Aljouf Province, Saudi Arabia; 2 Department of Biomedical Science, Faculty of Medicine and Health Sciences, Universiti Putra Malaysia, UPM Serdang, Selangor, Malaysia; 3 Genetics and Regenerative Medicine Research Group, Universiti Putra Malaysia, UPM Serdang, Selangor, Malaysia; 4 Department of Biotechnology, Bharath Institute of Higher Education and Research, Chennai, Tamil Nadu, India; 5 Department of Medical Microbiology and Parasitology, Universiti Putra Malaysia, UPM Serdang, Selangor, Malaysia; 6 Department of Biomedical Sciences, School of Medicine, Nazarbayev University, Nur-Sultan, Kazakhstan; 7 Department of Pharmacology, Hamidiye International Faculty of Medicine, University of Health Sciences, Uskudar, Istanbul, Turkey; Bharathidasan University, INDIA

## Abstract

Multiple matrix metalloproteinases have significant roles in tissue organization during lung development, and repair. Imbalance of proteinases may lead to chronic inflammation, changes in tissue structure, and are also highly associated to cancer development. The role of MMP20 is not well studied in lung organogenesis, however, it was previously shown to be present at high level in lung adenocarcinoma. The current study aimed to identify the functional properties of MMP20 on cell proliferation and motility in a lung adenocarcinoma in vitro cell model, and relate the interaction of MMP20 with other molecular signalling pathways in the lung cells after gaining tumoral properties. In this study, two different single guide RNA (sgRNAs) that specifically targeted on *MMP20* sites were transfected into human lung adenocarcinoma A549 cells by using CRISPR-Cas method. Following that, the changes of *PI3-K*, *survivin*, and *MAP-K* mRNA gene expression were determined by Real-Time Polymerase Chain Reaction (RT-PCR). The occurrence of cell death was also examined by Acridine Orange/Propidium Iodide double staining. Meanwhile, the motility of the transfected cells was evaluated by wound healing assay. All the data were compared with non-transfected cells as a control group. Our results demonstrated that the transfection of the individual sgRNAs significantly disrupted the proliferation of the A549 cell line through suppression in the gene expression of *PI3-K*, *survivin*, and *MAP-K*. When compared to non-transfected cells, both experimental cell groups showed reduction in the migration rate, as reflected by the wider gaps in the wound healing assay. The current study provided preliminary evidence that *MMP20* could have regulatory role on stemness and proliferative genes in the lung tissues and affect the cell motility. It also supports the notion that targeting MMP20 could be a potential treatment mode for halting cancer progression.

## 1.0 Introduction

Lung organogenesis studies have observed spatiotemporal expression of multiple matrix metalloproteinases (MMPs) and are tightly regulated. The lung is highly exposed to physical, chemical or biological insults. The resurgence of MMPs as a response to lung tissue injury implies that these molecules may protect and help to restore tissue structure and function by modulating immune response [1], and activating tissue repair processes [2]. Failure to reverse DNA damage incurred by certain agents, for example, nicotine in tobacco smoking can result in lung cell dysplasia [3, 4].

Lung adenocarcinoma resistant to epidermal growth factor receptor tyrosine kinase inhibitors *EGFR*-TKI is linked to *MMP1*, which is proposed to have a pivotal role in migration and invasion [5]. Additionally, *MMP2* and *MMP9* orchestrate invasion and metastasis in A549 cells and the expression of both proteins are significantly higher in lung adenocarcinoma compared with normal tissue surrounding the tumour [6, 7].

*MMP20* is present in the chromosome 11q22.3 and it was considered confined to the oral cavity, important for tooth formation [8, 9]. Much is unknown about the role of *MMP20* and its gene expression has been reported to maintain at same level throughout lung development [2]. However, a recent study showed that it is highly expressed in lung adenocarcinoma [10]. It is therefore, interesting to study the interaction of MMP20 with other signalling molecules involved in lung cell proliferation after gaining tumoral properties.

In this study, we hypothesized that *MMP20* participates in lung cell maintenance by regulating signalling pathways involved in cell stemness and proliferation. To prove this, we aimed to determine the downstream effect on the A549 lung adenocarcinoma cell proliferation following knock-down of *MMP20* gene expression by using CRISPR-Cas9 method. The fold changes of *phosphoinositide 3-kinase* (*PI3-K)*, *survivin* and *mitogen-activated protein kinase* (*MAPK)* gene expression were determined and compared with untreated cell line. In addition, the cell migration activity was also evaluated by wound healing assay.

## 2.0 Materials and methods

### 2.1 Cell culture

The human lung adenocarcinoma A549 cell line in vial format (P5) was obtained from the National Institute of Health (IMR), Selangor, Malaysia. The cells were thawed and cultured in 25 cm^2^ flasks in Roswell Park Memorial Institute 1640 Medium (RPMI 1640; Nacalai Tesque Inc.; Kyoto, Japan) supplemented with 10% Fetal Bovine Serum (FBS) (Gibco; USA) and 1% penicillin/streptomycin (Nacalai Tesque Inc.) at 37°C in an incubator with 95% air and 5% carbon dioxide (CO_2_). The cell growth was monitored and once it reached 80% confluency, the cells were subsequently sub-cultured into new flasks at a ratio of 1:3.

### 2.2 Guide RNA

Two pre-designed synthetic guide RNAs (sgRNA) targeting for human *MMP20* gene were purchased from Integrated DNA Technologies (IDT; Singapore). Hs.Cas9.MMP20.1.AA (5’-GGUCUAACUUCCCGGUGACU-3’) targets on the position of 102616920 on the positive strand, while Hs.Cas9.MMP20.1.AB (5’-GGGAUUCCUAUCCAUUCGAU-3’) targets on the position of 102610004 on the negative strand of the *MMP20* gene.

### 2.3 Transfection

One day before transfection, 1 x 10^4^ A549 cells were seeded into a 96-well plate and incubated at 37°C in a CO_2_ incubator. On the next day, two microcentrifuge tubes were prepared for lipofection experiment. In the first tube, 5 μl Opti MEM medium, 0.05 μl Cas9 protein, 1.5 μl MMP20 gRNA and 0.5 μl Lipofectamine Cas9 reagent were added and gently mixed. Then, the second tube containing 5 μl Opti MEM medium was prepared and added with 0.3 μl lipofectamine CRISPRMAX reagent. The second tube was incubated at room temperature (25°C) for 1 min. After one minute, the content of both tubes was mixed together and allowed to further incubate at room temperature for 15 min. Then, 10 μl of the complex mixture was gently added to each 96-well containing the cells. The plate was placed in the CO_2_ incubator for 2 days. After that, the culture medium was gently removed and the cells were rinsed with 50 μL of PBS. The cells were harvested for further downstream experiments. For non-transfected control group, the gRNA was replaced with deionized water.

### 2.4 RNA extraction and quantitative PCR (qPCR)

Total RNA was extracted from transfected and non-transfected A549 cells using the RNeasy® Mini kit (QIAGEN) according to the manufacturer’s instructions. The RNA was first reverse transcribed to cDNA (Takara Biotechnology Co., Ltd.; Dalian, China) before the expression levels of *PI3-K*, *survivin* and *MAP-K* were determined by quantitative RT-PCR using THUNDERBIRD® SYBR® qPCR Mix (Toyobo; Osaka, Japan). The reaction was performed on Light Cycler® 480 Real-Time PCR System (Roche Molecular Systems; California, USA), and the results were normalised with the expression of β-Actin (internal control). The primer pairs were synthesised by MyTACG Bioscience (Malaysia) and the sequences were described as follows: PI3-K (182 bp) forward: 5’-GGTGAAGCTCGTGTGTGGA-3’; reverse: 5’-GAAGACAGGGCTCCACTTCC-3’; survivin (103 bp) forward: 5’-CCAGACGATGACCCATGGAC-3’; reverse: 5’-TGAAGAACTCTGCCACCGTC-3’; MAP-K (187 bp) forward: 5’-CAGTTCTTGACCCCTGGTCC-3’; reverse: 5’-GTACATACTGCCGCAGGTCA-3’; β-actin (72 bp) forward: 5’-GACAGGATGCAGAAGGAGATCACT-3’; reverse: 5’-CTAAGGAGGAGCAATGATCTTGAT-3’. The relative mRNA expression was calculated according to the 2−ΔΔCt method.

### 2.5 Wound healing assay

Wound healing assay was performed to measure the migration potential of wild type A549 cells and transfected A549 cells. In brief, 1 x 10^6^ A549 lung cancer cells were seeded into a 12-well plate on Day 0 and incubated at 37°C and 5% CO_2_ for 24 h until confluency in the monolayer was achieved. With the help of a sterile micropipette tip, a scratch was made on the cell monolayer of all groups, then the wound width was observed at 40 x total magnification and pictures were taken. Then, transfections with the respective sgRNA (Hs.Cas9.MMP20.1.AA and Hs.Cas9.MMP20.1.AB) were performed according to the protocols described above. The transfected and non-transfected cells were incubated in a CO_2_ incubator for 48 h, the assay was performed in triplicate. After that, the micrograph pictures showing areas of scratch wound were taken. The length (distance) of cell migration was measured and analysed using ImageJ software and compared for all test groups. The formula below was used to calculate the rate of cell migration and the value was reported in the unit of nm per hour (nm/h):

$$\frac{RM=Wi-Wf}{t}$$

*RM* = Rate of cell migration (nm/h)

*Wi* = Initial wound width (nm)

*Wf* = Final wound width (nm)

*t* = Duration of migration (hour)

### 2.6 Cell viability determination

Post-transfection cell viability was evaluated by Acridine Orange/Propidium Iodide (AO/PI) double staining. The non-transfected and transfected cells were harvested and stained by mixing 10 μL of cellular pellet with a solution containing 10 μL AO/10 μL PI (1 mg/mL). The stained cells were gently pipetted onto a glass slide and covered with a cover slip before evaluation under a fluorescence microscope. The fluorescence microscopy was all performed within 30 min.

To determine possible cytotoxic effect of each component used in the transfection of gRNA into the cells, 3-[4, 5-methylthiazol-2-yl]-2, 5-diphenyl-tetrazolium bromide (MTT) (Ruibio: Zhejiang, China) assay kit was used to determine the number of viable A549 cells. In brief, 1 x 10^4^ A549 cells were seeded in a 96-well plate and incubated at 37°C for 24 h. The plate was divided into 6 test groups with triplicates containing reduced serum medium (Opti-MEM™ I, New York, USA). The test groups include the components used for transfection, i.e. Hs.Cas9.MMP20.1.AA sgRNA, Hs.Cas9.MMP20.1.AB sgRNA, Cas9 protein, Cas9 plus reagent, Lipofectamine CRISPRMAX, and blank (non-transfected cells). The plate was then incubated at 37°C for two days. After 48 h, 40 μl of MTT reagent was added to each well and incubated for 4 h at 37°C to allow development of reaction. At the end of reaction, the newly formed formazan crystals were dissolved with 100 μL DMSO and absorbance was recorded at 570 nm (with reference at 670 nm).

### 2.7 Statistical analysis

The quantitative data were stated as mean values ± standard deviation (SD). The differences in gene expression between transfected and non-transfected cells were determined by two-tailed t-test and confirmed to be significant if the *p*-value was less than 0.05.

## 3.0 Results

### 3.1 Transfected cells demonstrated lower expression of genes involved in survivability

To determine the knock-down effect of *MMP20* gene on A549 cell survivability, we used RT-PCR to quantitatively measure mRNA gene expression of *PI3-K*, *survivin* and *MAP-K* in the transfected cells. The changes in gene expressions after transfection were expressed as relative fold gene expression against non-transfected cells. This was performed by normalising the gene expression level with *ß-actin* as internal control and compared with non-transfected cells.

**Fig 1:**
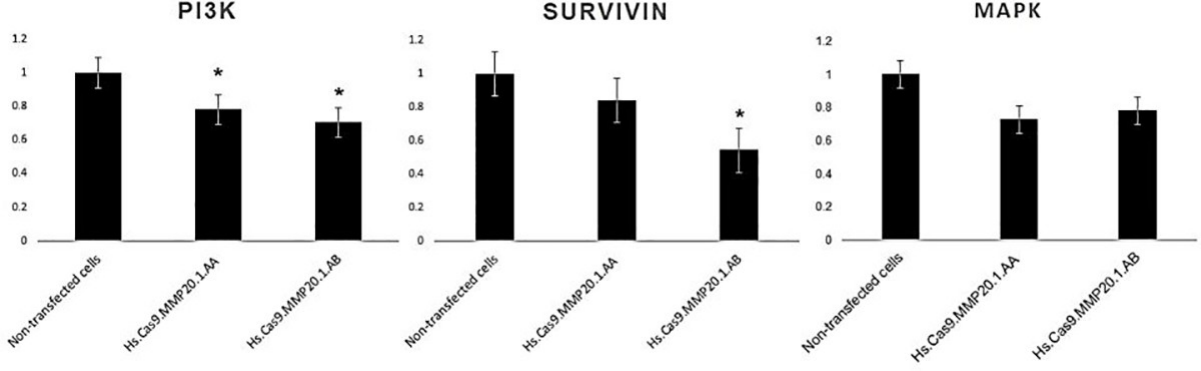
Fold changes in gene expression of *PI3-K* (A), *survivin* (B) and *MAPK* (C) in the test groups transfected with Hs.Cas9.MMP20.1.AA and Hs.Cas9.MMP20.1.AB gRNAs, respectively.

As represented in Fig 1A, cells transfected with Hs.Cas9.MMP20.1.AA and Hs.Cas9.MMP20.1.AB expressed *PI3-K* at 0.78 (*p* = 0.049) and 0.70-fold (*p* = 0.033) lower level than non-transfected cells, respectively. Meanwhile, Fig 1B also shows these transfected cell groups expressed *survivin* at 0.84 (*p*>0.05) and 0.54 (*p* = 0.017) fold lower than transfected cells. For *MAPK* gene expression (Fig 1C), the cells did not undergo significant level of changes after transfection with each of these single gRNA.

### 3.2 Knock-down of *MMP20* gene led to A549 cell apoptosis

To determine the effects of transfection on cell apoptosis, AO/PI double staining was performed after 48 h. The living nucleated cells would stain green, whereas dead nucleated cells would stain red.

**Fig 2:**
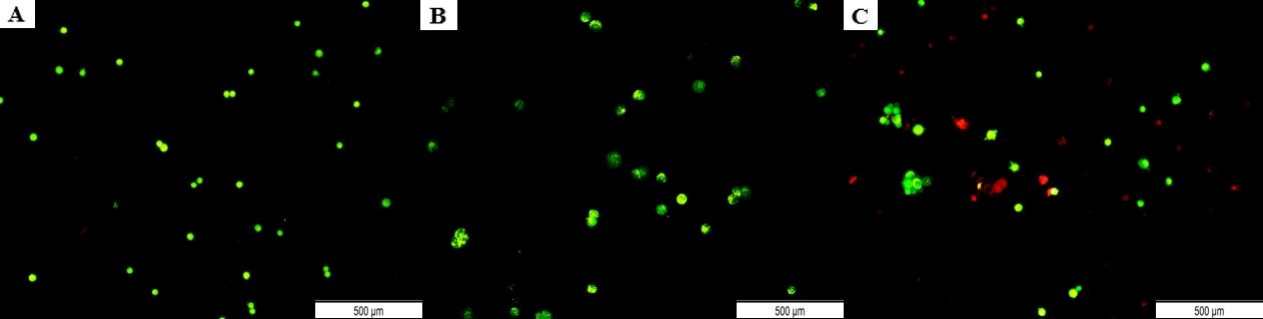
Microscopic observation of apoptosis in A549 cells 48 h post-transfection. AO/PI double staining was performed on non-transfected cells (A) and cells transfected with Hs.Cas9.MMP20.1.AA gRNA (B), and Hs.Cas9.MMP20.1.AA gRNA (C). All the images were taken with a magnification of *×*400. Scale 500 μm.

In contrast to non-transfected cells which showed high cell viability (Fig 2A), the cells transfected with Hs.Cas9.MMP20.1.AA gRNA presented early apoptotic changes with appearance of membrane blebbing (Fig 2B). Meanwhile, Hs.Cas9.MMP20.1.AB gRNA-transfected cells showed presence of many red nucleated cells, depicting an advanced stage of apoptosis (Fig 2C).

**Fig 3:**
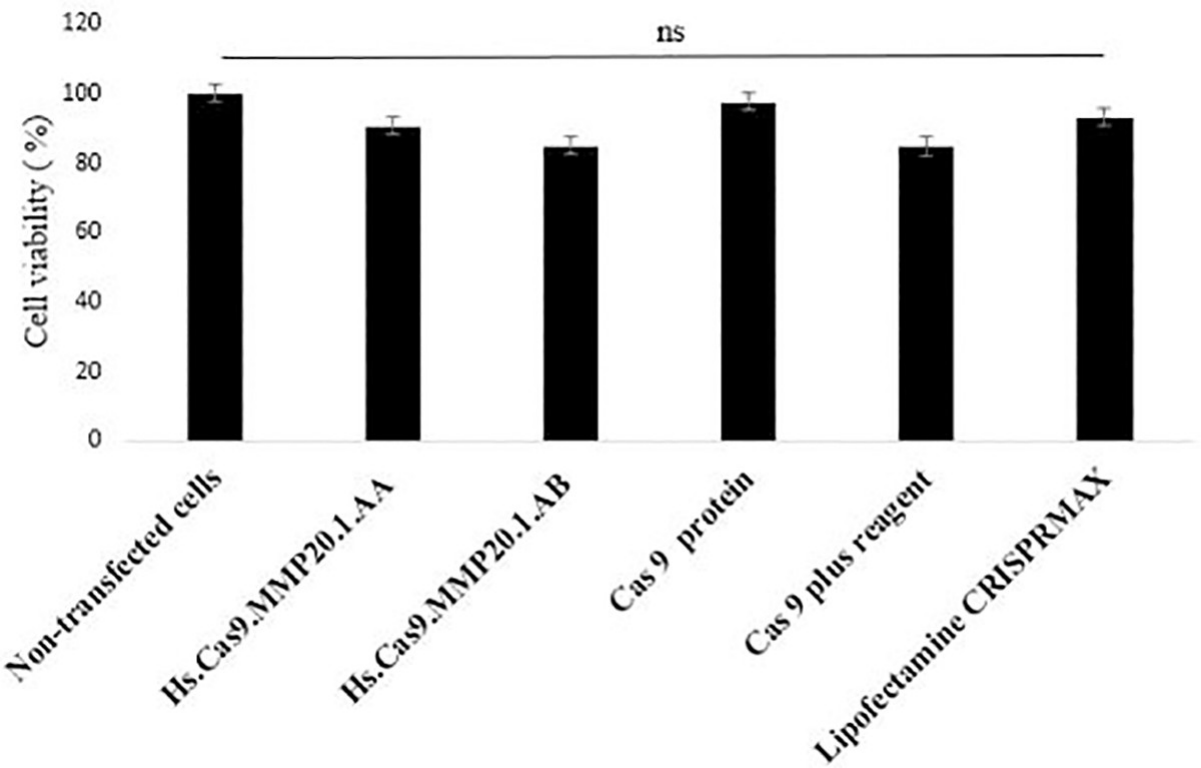
Cytotoxic effect of CRISPR-Cas9 reaction components on A549 cells lines. Cells were treated with individual reaction components for 48 h and the cell viability was determined by MTT assay. The data were expressed as mean values ± Standard Error of the Mean (S.E.M) of viable cells (n = 3).

To confirm that the cell apoptosis was not caused by the transfection method itself, the cytotoxicity of each transfection component (Hs.Cas9.MMP20.1.AA, Hs.Cas9.MMP20.1.AB, Cas9 protein, Cas9 plus reagent and Lipofectamine CRISPRMAX) following 48 h of incubation on A549 cell line was assessed through MTT assay. This experiment confirmed that the cell necrosis and apoptosis were specific to the *MMP20* knock-down by the delivery of the ribonucleoprotein complexes into the cells. The results did not indicate any significant increase in cell death when cells were transfected with Cas9 protein and compared with non-transfected control group (*p* > 0.05). (Fig 3).

### 3.3 Transfected cells showed a decrease in cell migration

To affirm that apoptosis event was a direct effect of successful knock-down of *MMP20* gene, a cell migration assay was performed. It was expected that the reduced expression of *MMP20* gene would lead to a decrease in cell motility.

**Fig 4:**
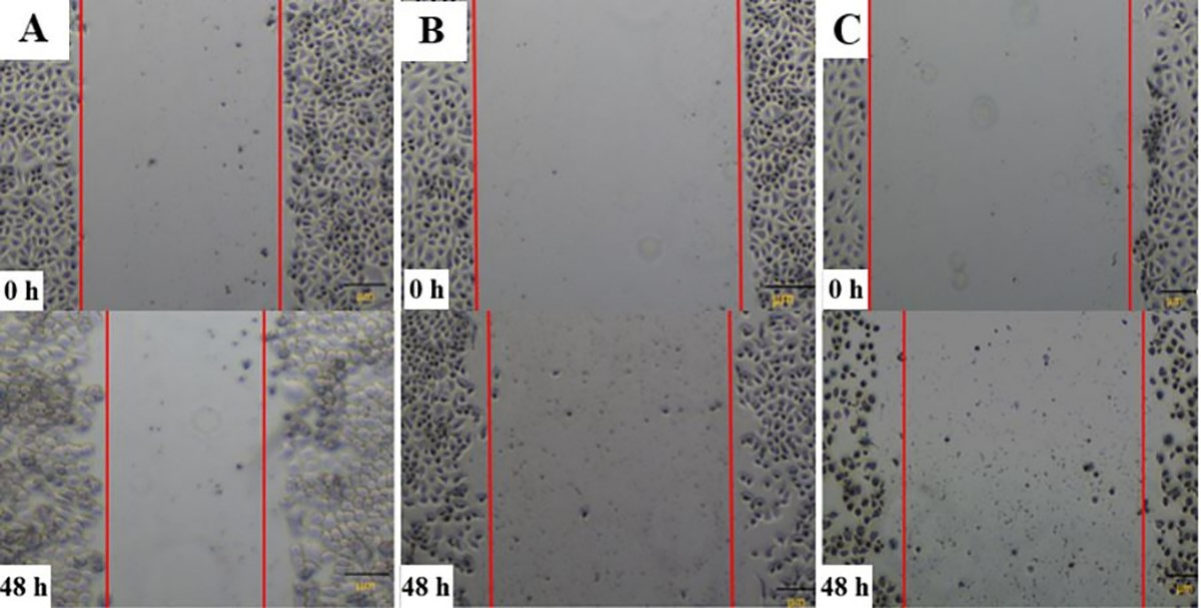
Comparison of wound gap between transfected and non-transfected cells. Wound healing assay was used to compare the ability of cells between the test groups to close the wound created on monolayer cells. The micrographs were taken for non-transfected (A), Hs.Cas9.MMP20.1.AA (B), and Hs.Cas9.MMP20.1.AB (C) transfected cells at 0 h and 48 h after creating the wound in the monolayer cells. All the images were taken with a magnification of *×*400.

**Fig 5:**
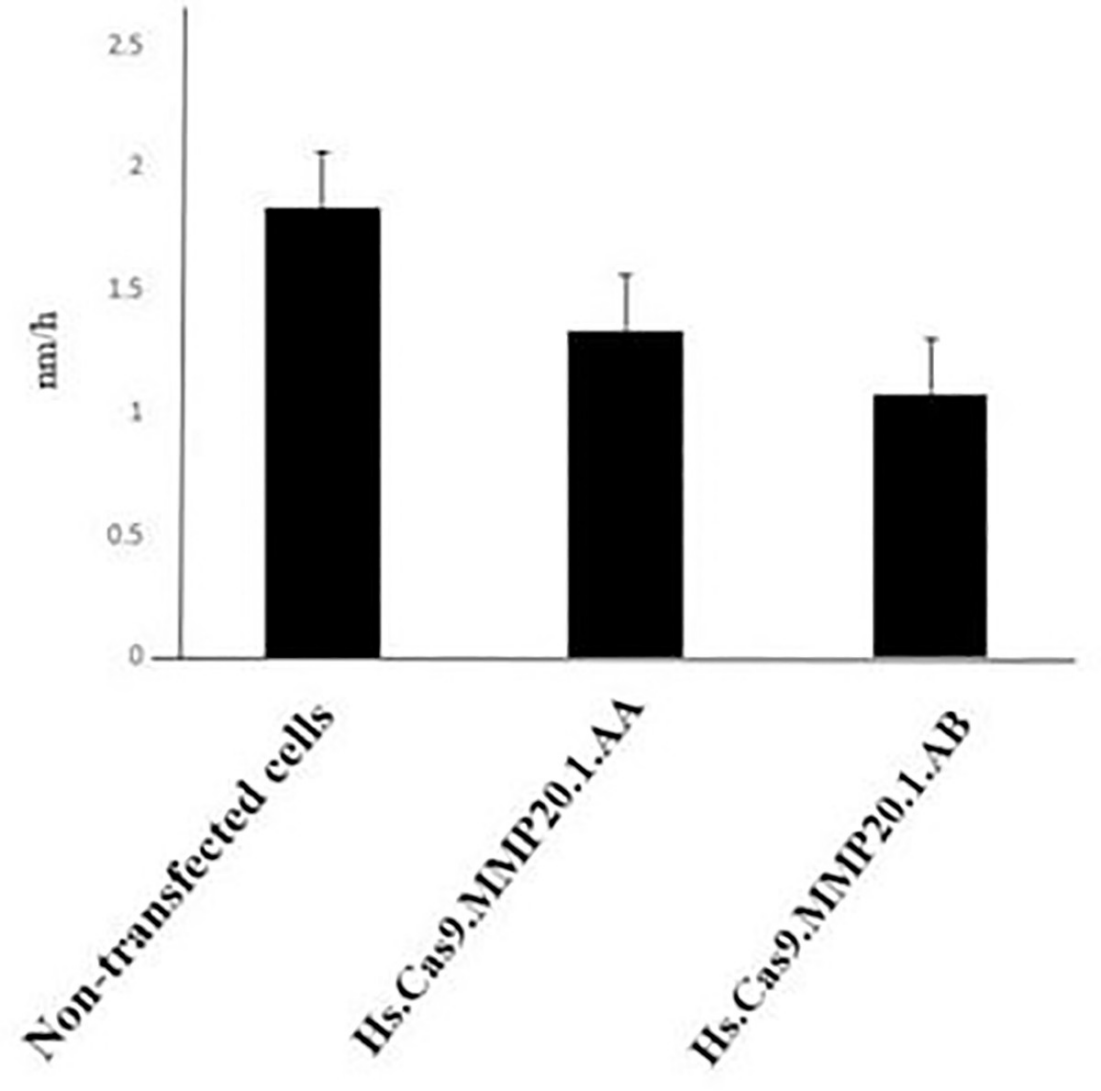
Rate of cell migration. After 48 h of transfection, the gap between the edges of the wound was analysed with ImageJ software, and the rate of cell migration was calculated and represented as the distance of cell migration within one hour (nm/h). The cells transfected with either Hs.Cas9.MMP20.1.AA or Hs.Cas9.MMP20.1.AB gRNA showed reduction in its migration rate when compared to non-transfected cells (n = 3).

The results presented in Figs 4 and 5 depicted the changes in cell migration activity in non-transfected, Hs.Cas9.MMP20.1.AA and Hs.Cas9.MMP20.1.AB gRNA-transfected cells, respectively. Fig 4 showed a wider wound in both transfected cell groups (Fig 4B and 4C) after 48 h compared to non-transfected cells (Fig 4A), indicating curbing of cell migration in transfected cell groups. After the analysis of the micrographs using ImageJ software, the rate of cell migration was decreased in Hs.Cas9.MMP20.1.AA and Hs.Cas9.MMP20.1.AB when compared to non-transfected cells (Fig 5). However, the decreases were not significant.

## 4.0 Discussion

The role of major MMPs is mostly associated to tissue patterning during organogenesis, repair and orchestration of immune response [2]. Previous reports have examined that *MMP20* levels remain unchanged during lung development, however, the expression was highly upregulated upon acquiring tumoral properties [10]. High secretion of MMP could confer metastatic activity to the cells, thus leading to spread of cancer cells to distant sites in the body [8, 11]. Thus, it is interesting to determine the interaction of MMP20 with other signalling molecules in lung adenocarcinoma and the cellular behavioural changes after MMP20 knock-down.

The current study demonstrated that knock-down of *MMP20* gene in A549 lung adenocarcinoma cells was accompanied by a reduction of cell survivability, as shown in the AO/PI stains (Fig 2) and migratory activities (Figs 4 and 5). Real-Time quantitative PCR demonstrated down-regulation of *PI3-K*, *survivin* and *MAP-K* genes in the cells (Fig 1). The results we reported herein should be considered in the light of some limitations. Firstly, the downregulation of *MAP-K* was not considered significant with each of the gRNAs. Secondly, the expression of *survivin* was significantly downregulated only by Hs.Cas9.MMP20.1.AB. Since the gRNAs targeted only at one site in *MMP20* gene, the DNA could have repaired themselves following the double strand breaks made by the Cas9 in complex with the gRNAs [12]. It is also possible that the transfection methods, in particular, the concentration and variant of Cas protein used in this study, could have resulted in such a finding. Further optimization of transfection methods may improve the significance in gene expression differences between the transfected and non-transfected cells.

Literature reviews have shown that *PI3-K* [13–16], *survivin* [17, 18] and *MAP-K* activation [19, 20] are essential for cell proliferation. It is also possible that inhibition of *MMP20* may inactivate the *WNT*/*ß-catenin* signalling pathway [19] leading to reduction in cell proliferation. In addition to that, *WNT* signalling pathway is also responsible to promote cell motility and invasion through extracellular matrix such as Matrigel [21]. One study showed that knock-down of *MMP20* gene expression in several ovarian cancer cell lines correlated with decreases in cell invasion abilities [22]. Taken together, it is rationale to speculate that *MMP20* may have modulatory properties on cell cycle that leads to uncontrolled cell proliferation in cancer development, in addition to its role in cell metastasis.

It is noteworthy that *PI3-K*/*AKT*/*c-myc* signalling pathway plays pivotal role in initial lung development, and maintenance of lung cell stemness vital for tissue repair in adults [23]. *C-myc* transcription factor is an important component in the Yamanaka factors required to reprogram somatic cells to pluripotent state [24]. This signalling pathway is re-activated when cells switch to aberrant cell type as in cancer formation [24]. It is unclear whether or not *MMP20* upregulation occurs after cells acquire tumoral properties, however, our study clearly showed that *MMP20* regulates *PI3-K*, and possibly *c-myc* gene activities. This is in agreement with previous report by Nikitakis *et al*., which demonstrated that *MMP20* has a governing role on the maintenance of cancer stem cell (CSC) in human oral cancer cells (OCCs) [25]. When *MMP20* was silenced, the CSC markers such as ALDH1, BM1, CD44, LGR4 and CD133 were down-regulated in human OCCs.

To our knowledge, the present findings are the first to report on direct changes in cellular behaviour of human lung adenocarcinoma, particularly on migratory activity, following inhibition of *MMP20* gene expression, and provides a link to molecular function of *MMP20* in cancer cell survivability. These findings implicate that *MMP20* might regulate numerous signalling pathways that promote cancer development and progression, and should be confirmed with a Next-Generation Sequencing study. Also, future efforts should explore targeting *MMP20* gene as a form of treatment for lung adenocarcinoma.

Current mode of treatment for lung cancer includes surgery, radio- and chemotherapy, and prescription of targeted inhibitor drugs such as cisplatin, bevacizumab, entrectinib, erlotinib and cetuximab, to the patients [10, 11, 26]. Despite that, these treatments resulted in poor prognosis and low five-year survival rate [27].

Since the discovery of CRISPR-Cas9 in bacterial immune system [28], CRISPR-Cas9 has been adapted into a powerful tool for cancer genomic research [29, 30]. Over the past decade, the CRISPR-Cas9 technology has become a popular technique for genome editing because it is rapid, cost-effective, precise and relatively easy to perform [31]. In lung cancer research, CRISPR-Cas9-mediated gene knockout of *Bcl-2 interacting cell death suppressor* (*BIS*) has demonstrated a substantial decrease in survival to cisplatin treatment in A549 cells [32]. Other than that, it has also been used to make modifications in somatic cells including stem and germline cells for correcting various diseases such as pulmonary diseases [33], gastrointestinal disorders [34, 35], haematological diseases [36–38] viral diseases [39–41], vector diseases [42, 43], autoimmune and inflammatory diseases [44, 45]. The safety of CRISPR-based therapy for precise gene targeting is currently evaluated in clinical trials [46], and may be potentially useful for cancer targeting.

## Conclusion

The knock-down of *MMP20* gene in human A549 lung adenocarcinoma could trigger cell death and decrease cell migration in the *in vitro* assay. *MMP20* is involved in the regulation of *PI3-K*, *survivin* and *MAP-K* gene expressions. Specific targeting on *MMP20* gene could potentially provide an efficient form of treatment to halt lung adenocarcinoma progression. More studies should be carried out in the future to evaluate the safety and efficiency of CRISPR-Cas technologies for knocking down *MMP20* gene in lung adenocarcinoma animal models.
